# Proanthocyanidin-Rich Fractions from Red Rice Extract Enhance TNF-α-Induced Cell Death and Suppress Invasion of Human Lung Adenocarcinoma Cell A549

**DOI:** 10.3390/molecules24183393

**Published:** 2019-09-18

**Authors:** Chayaporn Subkamkaew, Pornngarm Limtrakul (Dejkriengkraikul), Supachai Yodkeeree

**Affiliations:** 1 Department of Biochemistry, Faculty of Medicine, Chiang Mai University, Chiang Mai 50200, Thailand; subkamkaew@gmail.com (C.S.); ); 2 Anticarcinogenesis and Apoptosis Research Cluster, Faculty of Medicine, Chiang Mai University, Chiang Mai 50200, Thailand; 3 Center for Research and Development of Natural Products for Health, Chiang Mai University, Chiang Mai 50200, Thailand

**Keywords:** proanthocyanins, TNF-α, autophagy, invasion, lung adenocarcinoma

## Abstract

Tumor necrosis factor-alpha (TNF-α) plays a key role in promoting tumor progression, such as stimulation of cell proliferation and metastasis via activation of NF-κB and AP-1. The proanthocyanidin-rich fraction obtained from red rice (PRFR) has been reported for its anti-tumor effects in cancer cells. This study investigated the molecular mechanisms associated with PRFR on cell survival and metastasis of TNF-α-induced A549 human lung adenocarcinoma. Notably, PRFR enhanced TNF-α-induced A549 cell death when compared with PRFP alone and caused a G0-G1 cell cycle arrest. Although, PRFR alone enhanced cell apoptosis, the combination treatment induced the cells that had been enhanced with PRFR and TNF-α to apoptosis that was less than PRFR alone and displayed a partial effect on caspase-8 activation and PARP cleavage. By using the autophagy inhibitor; 3-MA attenuated the effect of how PRFR enhanced TNF-α-induced cell death. This indicates that PRFR not only enhanced TNF-α-induced A549 cell death by apoptotic pathway, but also by induction autophagy. Moreover, PRFR also inhibited TNF-α-induced A549 cell invasion. This effect was associated with PRFR suppressed the TNF-α-induced level of expression for survival, proliferation, and invasive proteins. This was due to reduce of MAPKs, Akt, NF-κB, and AP-1 activation. Taken together, our results suggest that TNF-α-induced A549 cell survival and invasion are attenuated by PRFR through the suppression of the MAPKs, Akt, AP-1, and NF-κB signaling pathways.

## 1. Introduction

Lung cancer is the most commonly diagnosed form of cancer and the primary cause of cancer-related mortality for males worldwide. It is also the second leading cause of cancer-related deaths among women globally [1]. Lung cancer is aggressive, and its treatment remains a difficult and challenging task for physicians [2]. Several research studies have indicated that long-term exposure to inhaled carcinogens has the greatest impact on increased risks of lung cancer [1,3,4,5]. Inhalation of such toxic air pollutants and microorganisms can cause lung injuries and chronic inflammation [6]. Chronic inflammation has been associated with cancer development. Many proinflammatory mediators, especially cytokines, chemokines, and prostaglandins, have been found to promote cancer proliferation, invasion, angiogenesis, and drug resistance [7].

A large number of studies have indicated that TNF-α displays a degree of potential in linking the molecules associated with inflammation and cancer. The data obtained from clinical studies have revealed that the expression level of TNF-α in the tumor tissues and serum samples obtained from patients with non-small cell lung cancer increased along with the clinical stage of the tumor [8,9]. TNF-α plays an important role in the process by binding itself to the tumor necrosis factor R-1 (TNFR-1). After the binding of TNF-α and TNFR-1, the receptor interacts with TRADD (TNFR1-associated death domain) to initiate the recruitment of receptor-interacting protein 1 (RIP1) and TNFR-associated factor 2 (TRAF2) [10]. These complex signaling lead to induce cancer cell survival, proliferation, and metastasis via upregulation of antiapoptotic (Survivin, XIAP, Bcl-xl, Bcl-2, and cFLIP), proliferative (cyclin D and cyclin B1), invasive (MMP-9, MT1-MMP, uPA, and uPAR), and angiogenic (VEGF, and COX-2) proteins by activating NF-κB, activator protein-1 (AP-1), and the mitogen-activated protein kinase (MAPKs) signaling pathway [11,12]. On the other hand, the binding of TNF-α and TNFR-1 can induce the program cell death that is involved with apoptosis by recruiting TRADD-FADD and caspases-8. In fact, TRADD and caspase-8 complex are assembled over a delayed period of time when compared with TRAF2 and RIP1, which results in a sufficient amount of time needed to activate survival signaling. Therefore, the expression of anti-apoptotic proteins and caspase inhibitors, including Bcl-2, Bcl-xl, xIAP, and cFLIP, would be elevated prior to caspase 8 activation [13,14]. Thus, the blockade of TNF-α-induced survival signaling can lead to an increase in the sensitivity of TNF-α-induced cell death. Moreover, many studies have shown that TNF-α expression results in the induction of multiple autophagy markers in breast cancer cells, lung cancer cells, and Ewing’s sarcoma cells [15]. A novel function of anti-apoptotic proteins, such as cFLIP, survivin, Bcl-2, and Bcl-xl, that serve as autophagy inhibitors, have been reported in various cells [16,17]. Downregulation of these antiapoptotic proteins could enhance TNF-α-induced cancer cell death via autophagy and apoptosis. Accordingly, the efficient agents that can suppress TNF-α-induced cancer cell progression could be an important part of an attractive and alternative form of cancer therapy. 

Proanthocyanidins, also known as condensed tannins, are a class of polymeric phenolic compounds that consist mainly of catechin, epicatechin, gallocatechin, and epigallocatechin units [18]. Recently, our previous findings have demonstrated that proanthocyanidin-rich fractions derived from red rice (PRFR) inhibited inflammation in LPS-treated Raw 264.7 cells via suppression of the AP-1, NF-κB, and MAPKs signaling pathways [19]. Moreover, PRFR reduced human fibrosarcoma, HT1080 cells and breast adenocarcinoma, MDA-MB-231 cells invasion via inhibition of the expression of invasive proteins [20]. Furthermore, PRFR suppressed cell proliferation in human hepatocellular carcinoma, HepG2 cells via the downregulation of survival proteins and induced cell apoptosis by enhancing active apoptotic proteins [21].

However, the effect of PRFR on TNF-α-induced cancer progression has not yet been clarified. Therefore, the purpose of this study was to investigate whether PRFR exerts anticancer effects through suppression of the TNF-α-induced expression of the survival and metastasis proteins by inhibiting the MAPKs, NF-κB, and AP-1 signaling pathways in A549 human lung adenocarcinoma cells.

## 2. Results

### 2.1. PRFR Enhanced TNF-α-Induced Cytotoxicity in A549 Lung Adenocarcinoma Cells 

**Figure 1:**
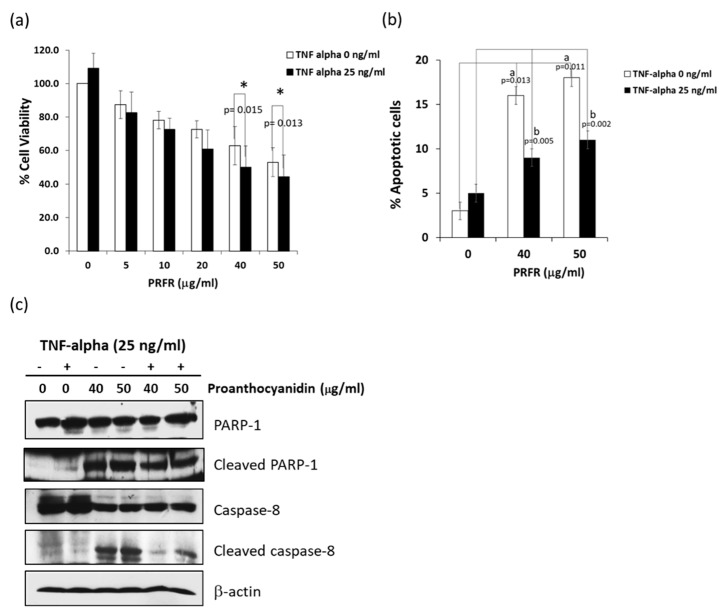
PRFR-enhanced tumor necrosis factor-alpha (TNF-α)-induced cytotoxicity in A549 lung adenocarcinoma cells. (**a**) A549 cells were preincubated with different concentrations of PRFR for 4 h and then co-treated with 25 ng/mL of TNF-α for 24 h. (**b**) Cell apoptosis was determined by Guava Nexin and analyzed by Guava^®^ easyCyte Flow Cytometer to detect the apoptotic cell population. (**c**) The apoptotic proteins were detected by western blotting using the antibodies to caspase-3, caspase-8, and PARP-1. The data are presented as mean ± S.D. with * *p* < 0.05, and ** *p* < 0.01 when compared with the PRFR alone, ^a^ *p* < 0.05 when compared with the control group, and ^b^ *p* < 0.01 when compared with the TNF-α alone.

The cytotoxicity of PRFR was examined by using trypan blue staining assay. Treatment of A549 cells with PRFR (0–50 μg/mL) for 24 h significantly reduced the viability of the cells in a dose-dependent manner. In particular, treatment of the cells with 40 and 50 µg/mL of PRFR decreased cell viability to 63.0% and 54.6%, respectively. However, a combination treatment of the cells with TNF-α (25 ng/mL), PRFR 40, and 50 μg/mL reduced cell viability to 42.3% and 36.5%, which significantly increased cytotoxicity to greater levels than in the treatment with PRFR alone (Figure 1a). Next, we investigated whether the enhancement activity of PRFR on TNF-α-induced cell death was associated with apoptosis by employing Annexin V-PI staining assay. The results indicate that treatment with PRFR alone at 40 and 50 μg/mL induced the apoptotic population from 3% to 16% and 18%, respectively (Figure 1b). However, co-treatment of PRFR at 40 and 50 μg/mL and TNF-α significantly induced the apoptotic population to a degree that was less than with the treatment of PRFP alone. To confirm whether or not apoptosis is the main cause of PRFR enhanced TNF-α induced cell death, the level of the apoptotic signaling pathway proteins was investigated by including cleaved caspase-8 and PARP-1. As shown in Figure 1c, the levels of caspase-8 and PARP-1 in a combination treatment were lower than for PRFP alone. These results indicate that PRFR could enhance the cytotoxicity effect of TNF-α; however, this result was not limited to the apoptotic pathway.

### 2.2. PRFR Potentiates TNF-α-Induced Autophagy 

**Figure 2:**
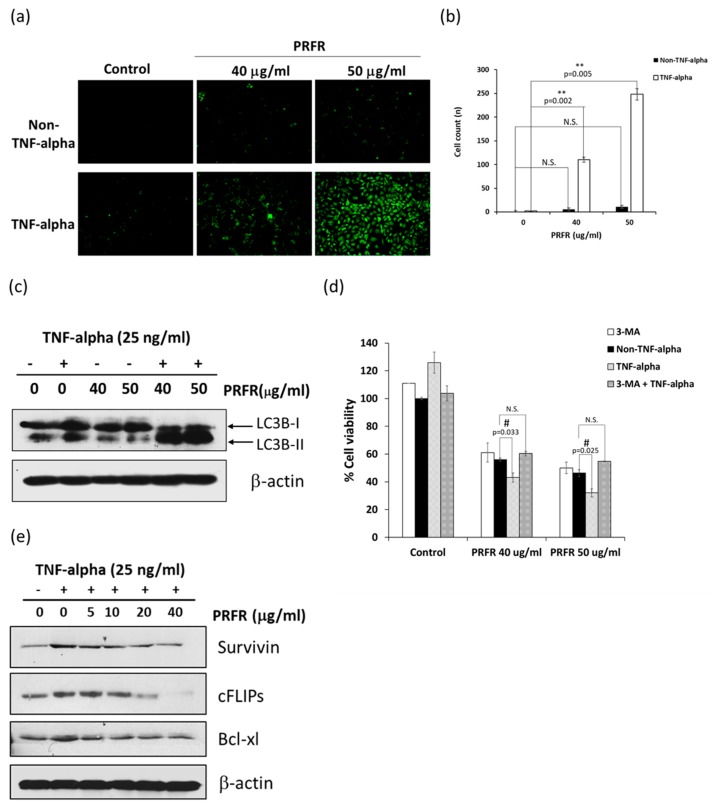
PRFR enhanced TNF-α-induced autophagic cell death in A549 cells. (**a**,**b**) A549 cells were stained with monodansylcadaverin (MDC) after being preincubated with 40 and 50 μg/mL PRFR and then co-treated with 25 ng/mL of TNF-α for 24 h. The data are presented in bar graphs (**b**). (**c**) The expression of autophagosome proteins (LC3B) was detected by western blot analysis using antibodies against LC3B. (**d**) A549 cells were preincubated with 1.5 mM of 3-MA for 1 h and then treated with 40 and 50 μg/mL PRFR and 25 ng/mL of TNF-α for 24 h, and the cell viability was determined using trypan blue assay. (**e**) The expression of survival proteins was detected by western blot analysis using the antibodies against survivin, cFLIPs, and Bcl-xl. Data from a typical experiment are depicted here, while similar results were obtained from three independent experiments. The data are presented as mean ± S.D. with ** *p* < 0.01 when compared with the TNF-α alone, and ^#^ *p* < 0.05 when compared with control group (N.S., not significant).

TNF-α-induced cell death occurred via the apoptosis pathway, but also stimulated autophagy cell death. Therefore, we investigated whether the enhancement activity of PRFR on TNF-α-induced cell death was involved with autophagy. The autophagy vacuoles were labeled by Monodansylcadaverin (MDC) fluorescent staining and analyzed them with a fluorescent microscope. Co-treatment of PRFR and TNF-α significantly increased the number of autophagy vacuoles in A549 cells when compared with TNF-α alone. However, PRFR alone did not induce autophagy vacuoles (Figure 2a,b). To further confirm PRFR mediated autophagy cell death in TNF-α-induced A549 cells, the expression level of LC3B-II, a credible marker of the autophagosome [22,23], was assayed by western blot analysis. Combination treatment with PRFR and TNF-α increased the expression levels of LC3B-II when compared with TNF-α alone, whereas PRFR alone had no effect (Figure 2c). To verify that autophagy plays a major role in the process of PRFR enhancement of TNF-α-induced cell death, the cells were co-treated with 3-MA (autophagy inhibitor), TNF-α, and PRFR for 24 h, and the cell viability was then analyzed. As shown in Figure 2d, combination treatment with 3-MA, PRFR, and TNF-α did not significantly reduce the cell viability when compared with PRFR alone. This results indicated that 3-MA attenuated the enhancement effect of PRFR on TNF-α-induced cell death by reversing the percentage of cell viability to the same level of treatment with PRFR alone (Figure 2d). In addition, the modulation effect of PRFR on the autophagy regulated proteins was determined. The results presented in Figure 2e. show that the induction of survivin, cFLIPs, and Bcl-xl by TNF-α were reduced by PRFR in a dose-dependent manner. Taken together, these results indicate that PRFR could enhance TNF-α-induced A549 cell death via the autophagy and apoptosis pathways.

### 2.3. Effect of PRFRon TNF-α-Induced Cell Proliferation

**Figure 3:**
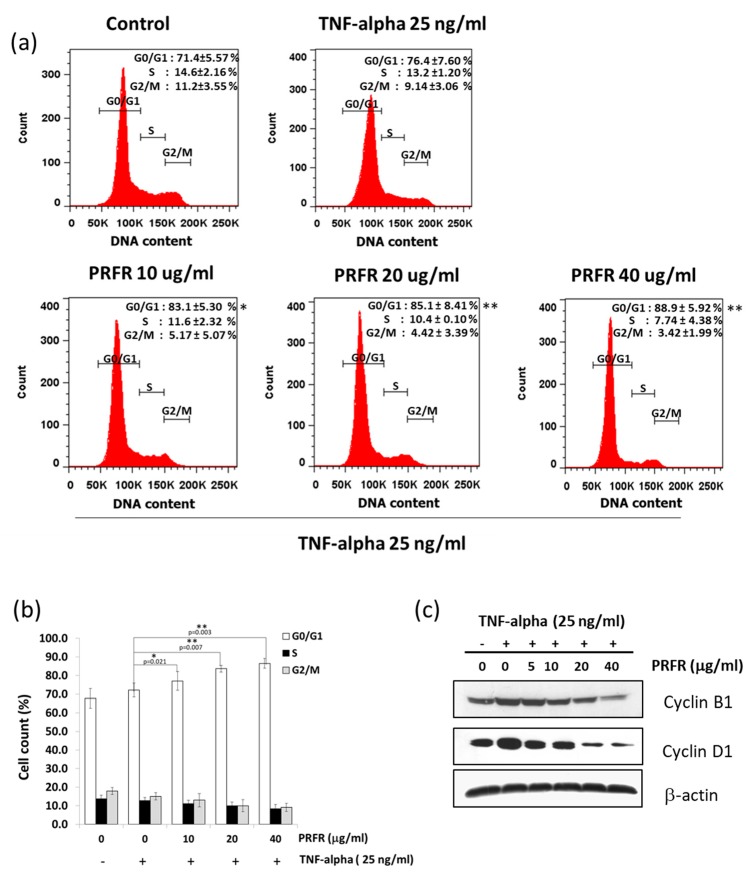
Effect of PRFR on TNF-α-induced cell proliferation. A549 cells were preincubated with various concentrations of PRFR for 4 h and then co-treated with 25 ng/mL of TNF-α for up to 24 h (**a**,**b**) cell cycle was determined by PI staining and analyzed by flow cytometry to detect the cell cycle arrest. The data present in a bar graph (**b**). (**c**) The expression of proliferative proteins was detected by western blot analysis using the antibodies against cell proliferation proteins (cyclin D1). Data from a typical experiment are depicted here, while similar results were obtained from three independent experiments. The data are presented as mean ± S.D. with * *p* < 0.05 and ** *p* < 0.01 when compared with the TNF-α alone.

TNF-α plays an important role in cancer cell proliferation by inducing the expression of proliferative proteins. The effect of PRFR on TNF-α-induced cell proliferation was examined by using PI staining. To determine the anti-proliferative effects of PRFR, A549 cells were pretreated with PRFR (10–40 μg/mL) and then treated with 25 ng/mL of TNF-α. As is shown in Figure 3a,b, the percentages of the G0/G1 phase of the cells receiving the combination treatment with TNF-α and PRFR at 10, 20, and 40 μg/mL, significantly increased from 76.4% to 83.1%, 85.1%, 88.9%, respectively when compared with those of the TNF-α treatment alone. The manner in which TNF-α induced was examined the expression levels of cyclin D1, which are G0/G1 cell cycle regulatory proteins. As is shown in Figure 3b, TNF-α induced the expression levels of cyclin D1 was decreased when the cells were treated with PRFR at 20 and 40 μg/mL.

### 2.4. PRFR Inhibited TNF-α-Induced A549 Cell Invasion and Migration

**Figure 4:**
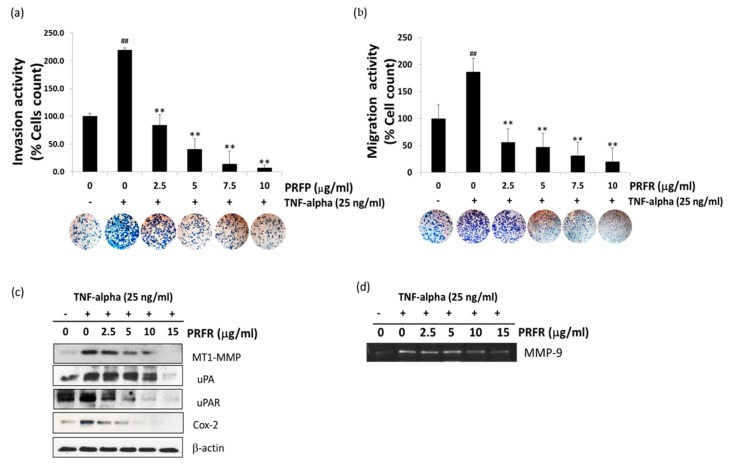
PRFR inhibits TNF-α-induced A549 cell invasion and migration. The matrix gel was coated on the upper surfaces of the membrane filters for invasion assay (**a**) and the gelatin was then coated for migration assay (**b**). Different concentrations of PRFR (0–10 μg/mL) with 1.25 × 10^5^ cells of the A549 cells were seeded into the upper chamber in DMEM serum-free medium, and the lower chamber was filled with 25 ng/mL of TNF-α. After 24 h of incubation, the migrated cells on the lower surface of the filter were determined. After co-treatment with PRFR and TNF-α for 24 h, whole-cell extracts were prepared and analyzed by western blot analysis using antibodies against metastatic proteins (MT1-MMP, uPA, uPAR, and COX-2) (**c**). The culture supernatants of the treated cells were collected, and the secretions of MMP-9 were analyzed by gelatin zymography (**d**). The data are presented as the mean ± S.D. of three independent experiments. Notably, (**a**,**b**) sample groups were found to be significantly different from the TNF-α-treated group (** *p* < 0.01) and the TNF-α alone compared with the control group (^##^ *p* < 0.01).

TNF-α plays a crucial role in lung cancer cell invasion. Therefore, the effect of PRFR on TNF-α-induced A549 cell invasion and migration was evaluated. The Figure 4a showed TNF-α efficiently induced A549 cell invasion through the basement membrane by 2.3-fold when compared with the control. However, in the presence of PRFR, TNF-α-induced invasion of A549 cells was significantly inhibited. Moreover, TNF-α also stimulated A549 cell migration by almost two-fold, while PRFR suppressed this activity. TNF-α promoted cancer cell metastasis by upregulating invasive proteins. Therefore, the effect of PRFR on TNF-α-induced proteins was examined that are involved in cancer cell invasion including MT1-MMP, uPA, uPAR, Cox-2, and MMP-9. As is shown in Figure 4c, TNF-α dramatically induced the expression levels of MT1-MMP, uPA, uPAR, and Cox-2 proteins after 24 h, while treatment of the cells with PRFR (0–15 μg/mL) prevented the TNF-α induced expression of these proteins in a dose-dependent manner. Next, the gelatin zymography assay was used to examine the inhibitory effects of PRFR on TNF-α-induced MMP-9 secretions. As is shown in Figure 4d, TNF-α-induced MMP-9 secretions were significantly inhibited in the presence of 10–15 μg/mL of PRFR.

### 2.5. Effect of PRFR on TNF-α-Induced NF-κB and AP-1 Activation

**Figure 5:**
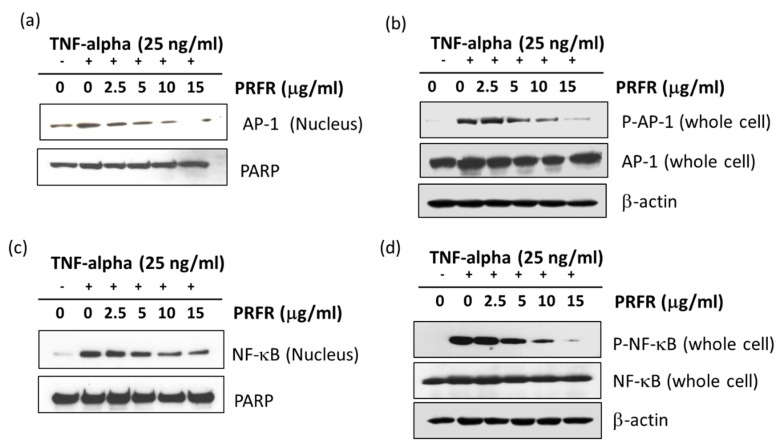
Effect of PRFR on TNF-α-induced NF-κB and activator protein-1 (AP-1) activation. A549 cells were pretreated with PRFR and induced with TNF-α 25 ng/mL for 1 h. The nucleus-extracted fraction was prepared in order to detect c-Jun (AP-1) (**a**) and p65 (NF-κB) levels (**c**) by western blot analysis. A549 cells were pretreated with PRFR for 12 h and induced with TNF-α 25 ng/mL for 15 min. The whole cell lysate was prepared for measurement of the phosphorylated and non-phosphorylated forms of AP-1 and NF-κB (**b**,**d**). β-Actin and PARP were used as internal loading control proteins in the cytoplasm and nucleus, respectively. Data from a typical experiment are depicted here, while similar results were obtained from three independent experiments.

NF-κB and AP-1 transcription factors are involved in cancer cell progression. The expression of survival, anti-apoptotic, autophagy, invasive, and angiogenesis genes are controlled by NF-κB and AP-1 transcriptional activity. To investigate whether PRFR affected TNF-α-induced NF-κB and AP-1 activation, nucleus translocation and phosphorylation of NF-κB and AP-1 were determined. As is shown in Figure 5a,b, TNF-α enhanced the nucleus translocation and phosphorylation of c-Jun (AP-1). PRFR could inhibit TNF-α-induced AP-1 translocation and blocked the TNF-α-induced phosphorylation of AP-1 in a dose-dependent manner. Next, the regulation of PRFR on the transcription activity of NF-κB was tested. The co-treatment with PRFR and TNF-α decreased TNF-α-induced nuclear translocation of p-65 (Figure 5c) and the phosphorylation of p65 at ser536 (Figure 5d) in a dose-dependent manner. The data indicate that PRFR can suppress the TNF-α-induced transcriptional activity of AP-1 and NF-κB.

### 2.6. Effect of PRFR on TNF-α-Induced MAPK, Akt, and IκB-α Signaling Pathways

**Figure 6:**
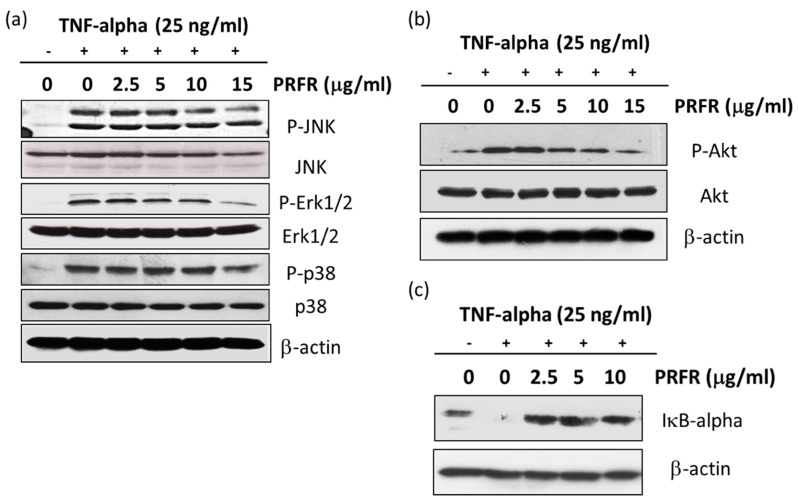
Effect of PRFR on TNF-α-induced MAPK, Akt, and IκB-α signaling pathways. A549 cells were pretreated with PRFR and induced with TNF-α 25 ng/mL for 15 min. The whole cell lysate was prepared for measurement of phosphorylated and non-phosphorylated forms of JNK, Erk1/2, p-38 (**a**), and Akt (**b**) by western blot analysis. The whole cell lysate was also used to determine TNF-α-induced IkB-α degradation (**c**) by western blot analysis. Data from a typical experiment are presented here, while similar results were obtained from three independent experiments.

TNF-α-activated MAPKs, Akt, and IκB-α signaling pathways have been involved in tumor progression via AP-1 or NF-κB transcriptional activities. Therefore, the effects of PRFR on the TNF-α-induced activation of IκB-α, Akt and MAPKs, including Erk1/2, p38, and JNK, were investigated by western blot analysis. As is shown in Figure 6a,b, TNF-α stimulated the phosphorylation of p38, JNK, and Erk1/2. Additionally, PRFR at 10 and 15 μg/mL inhibited the TNF-α-induced phosphorylation of the JNK and Erk1/2 signaling pathways, whereas, PRFR at 15 μg/mL reduced TNF-α induced phosphorylation of the p38 signaling pathway. On the other hand, the levels of the phosphorylated forms of Akt were induced by TNF-α. The level of TNF-α-induced phosphorylation of Akt was suppressed by PRFR in a dose-dependent manner, while the non-phosphorylation of Akt had no effect. Moreover, NF-κB activation by TNF-α was mediated via NIK and IKK, resulting in IκB-α degradation. In order to examine the effects of PRFR on IκB-α activity, PRFR affected the degree of TNF-α induced IκB-α degradation was determined. As is shown in Figure 6c, PRFR effectively blocked TNF-α-dependent IκB-α degradation.

## 3. Discussion

Proanthocyanidins are oligomers and polymers of flavanol-3-ol which are found in various fruits, vegetables, and cereals. Notably, they are present in foods such as grape seeds, blackberries, and red rice [18,24]. Our previous study revealed that PRFR exhibited anti-cancer activities by inducing HepG2 hepatocarcinoma cell apoptosis and inhibiting MDA-MB-231 breast cancer cell invasion [20,21]. Despite its various pharmacological activities, the molecular mechanism of PRFR on the anti-tumor effects in A549 lung adenocarcinoma cells has not been elucidated. In this study, we investigated whether PRFR could sensitize TNF-α-induced cell death in lung A549 cancer cells and then act as a potent inhibitor of TNF-α-induced A549 cell metastasis. Molecular mechanisms of this phenomena have been elucidated.

The interaction between TNF-α and TRFR-1 can trigger survival or death signaling pathways. It has been reported that most cancer cells are resistant to TNF-α-induced cell death via increased expressions of survival proteins through the induction transcriptional activity of NF-κB. Thus, a blockade of the activation of the survival signaling pathways may lead to an increase in sensitivity in TNF-α-induced cell death [12]. This study was the first to report that PRFR sensitized A549 lung adenocarcinoma cells to TNF-α-induced cell death. TNF-αpromotes cancer cell death by induction apoptosis, necroptosis, and autophagy depending on the condition of the cells. TNF-α induced cell apoptosis via the intrinsic and extrinsic pathways. The results of this study demonstrate that PRFR alone enhanced A549 cell apoptosis with increased caspase-8 activation while inducing PARP cleavage. However, a combination of PRFR and TNF-α induced A549 to apoptosis to a lesser degree than PRFR alone and also revealed a partial effect on caspase-8 activation and the level of the cleaved PARP. This would suggest that apoptosis is more than a mechanism for PRFR to enhance TNF-α-induced A549 cell death.

Autophagy has been extensively reported to play a critical role in the control of cell proliferation, differentiation, and cell death. Autophagy is a highly regulated and fundamental cellular homeostatic process, in which cytoplasmic material is delivered and organelles convert to lysosomes via double membrane vesicles called autophagosomes for degradation. Autophagy is activated in response to various forms of cellular stress, including starvation, hypoxia, radiation, and inflammation [25,26]. Many studies have shown that TNF-α-induced autophagic cell death occurs in various cancer cell types including breast cancer, hepatoma and ovarian cancer [27,28]. Therefore, autophagy is considered a potential pathway in the treatment of cancer. Many natural drug molecules, such as curcumin, celastrol, and bufalin, play important roles in tumor inhibition by inducing autophagy. Thus, PRFR was examined whether it can enhance TNF-α-induced A549 cell death via autophagy cell death. Co-treatment of PRFR and TNF-α led A549 cells to autophagy by accumulating autophagosomes and upregulating the expression of LC3B-II proteins. Whereas, PRFR alone did not induce autophagy, LC3s proteins were found to be a structural protein of autophagosome membranes. The conversion of a soluble form of LC3B-I to LC3B-II is often used to demonstrate active autophagy. To confirm that autophagy is a major process of PRFR in the enhancement of TNF-α induced cell death by using 3-MA as an autophagy inhibitor. The obtained results indicate that using 3-MA could reverse the enhancement effect of PRFR on TNF-α-induced cell death, which would indicate that the way in which PRFR enhanced the cytotoxicity effect of TNF-α was due to autophagy cell death. Recent findings have revealed a novel function of anti-apoptotic proteins, such as FLIP, survivin, Bcl-2, and Bcl-xl, as negative regulators of autophagy. FLIP has been shown to inhibit LC3 lipidation by competitive interaction with ATG3, which in turn blocks autophagy [16]. Moreover, Zhu J., et al. have shown that the inhibition of survivin through the use of siRNA enhanced autophagy by upregulating Beclin-1 [29]. Therefore, in order to explain the mechanism by which PRFR sensitizes TNF-α-induced autophagy, the modulatory effect of PRFR on TNF-α induced FLIP, Bcl-xl, and survivin expression levels was examined. It was found that the levels of FLIP, Bcl-xl and survivin were reduced by PRFR. Together, these results suggest that the manner in which PRFR enhanced TNF-α induced cell death was at least in part accomplished by down regulating FLIP, Bcl-xl and survivin, which then led to autophagic cell death.

Moreover, the results of this study indicate that PRFR can suppress cell proliferation by blocking cell cycle progression in the G1 phase. TNF-α is known to stimulate transcriptions of Cyclin D1, Cyclin E, and Cyclin B1 in order to accelerate the progression of the cell cycle. Cyclin D1 is a key regulator of the G1 checkpoint control [30]. This finding is consistent with our observation that PRFR suppressed TNF-α-induced expression of cyclin D1. This result suggests that PRFR reduced TNF-α induced cell proliferation by inhibiting the expression of cyclin proteins. 

The degradation of ECM and the components of the basement membrane through which proteases are the key steps of cancer cell invasion and metastasis. Of these proteases, MMPs such as MMP-9, MT1-MMP, and uPA are thought to play an important role in cancer invasion. Furthermore, COX-2 has been implicated in metastasis, and its overexpression can enhance cellular invasion, proliferation, and induce angiogenesis [31,32,33]. Previous observations have indicated that TNF-α is an inducer for the invasion and metastasis of A549 cells. These results clearly demonstrate that PRFR inhibits the TNF-α-induced invasion and migration of A549. Moreover, PRER reduced the levels of TNF-α-induced expression of invasive genes, including MMP-9, MT1-MMP, uPA, uPAR, and Cox-2, in the A549 cells.

NF-κB and AP-1 are major key players in TNF-α-mediated tumor progression. NF-κB regulates the expression of the survival gene products cIAP, Bcl-2, Bcl-xl, and FLIP, along with the proliferation of gene products cyclin B1 and cyclin D1, and the invasion of gene products uPA, COX-2, MMP-9, and MT1-MMP, which are known to be induced by TNF-α [31,34,35]. Furthermore, it has been reported that TNF-α could induce autophagy in cancer cells when NF-κB signaling is inhibited [36,37]. A common form of NF-κB is a heterodimer consisting of p50/p65. NF-κB is normally retained in the cytoplasm through interaction with its inhibitor IκB. Upon TNF-α stimulation, IκB-α is catalyzed for phosphorylation by IκB kinase (IKK) leading to IκB-α degradation and allowing for the nuclear translocation of NF-κB, which promotes the transcription of the corresponding genes. Therefore, we have determined the activity of PRFR on TNF-α can induce the degradation of IκB-α and the translocation of NF-κB activity. Our results demonstrated that PRFR prevented degradation of IκB-α and reduced NF-κB activity by inhibiting TNF-α-induced p65 phosphorylation and translocation to the nucleus of the cells. AP-1 has been implicated in regulating cancer cell survival and proliferation. AP-1 also controls the gene expression values of MMP-9, MT1-MMP, Cox-2, uPA, and uPAR. Here, the activation of AP-1 was investigated by observing the phosphorylation and translocation of c-Jun in TNF-α treated cells. In this study, PRFR inhibited TNF-α induced c-Jun phosphorylation and translocation to the nucleus of the A549 cells. This result was in accordance with the findings of an investigation conducted by Qiao Y et al., which also demonstrated that the suppression of AP-1 signaling can potentiate TNFα-induced cell death and inhibit cancer cell invasion [34]. Based on the above-mentioned results, we suggest that PRFR could decrease the level of expression of survival and metastasis proteins by the inhibition of AP-1 and NF-κB activation and are also in agreement with inhibition of AP-1 and NF-κB by epigallocatechin gallate reduced cancer cells survival and metastasis [38,39].

It is accepted that the activation of the MAPKs or Akt signaling pathways is important for regulating survival and metastasis in a variety of cancer cells. TNF-α is bound to the TNF-α receptor-1 which induces NF-κB activation by activating the MAPKs, Akt, and IKK signaling pathways. Moreover, the activation of MAPKs and Akt are important for regulating AP-1 activity. MAPKs are known to be serine/threonine kinase and are composed of several subgroups, such as ERK1/2, JNK, and p38 [35]. It is generally demonstrated that MAPKs signaling pathways regulate metastasis and survival in a variety of cancer cells. Accumulated evidence indicates that the Akt and MAPKs signaling pathways are involved with autophagy. The AKT/mTOR signaling pathway is one of the survival regulatory pathways in both normal and cancer cells, and it can negatively regulate autophagy [40]. Therefore, the experiments were performed to determine whether PRFR regulates TNF-α in order to stimulate the activity of MAPKs and Akt. Our results show that PRFR prevented the phosphorylation of p38, ERK, JNK, and Akt. These results are consistent with those of previous reports which have found that using the inhibitors of the PI3K/Akt and MAPK signaling pathways causes cell death and is associated with autophagy, apoptosis and a reduction in the invasive properties of cancer cells.

## 4. Materials and Methods 

### 4.1. Chemicals and Reagents

Dulbecco’s Modified Eagle Medium (DMEM), trypsin and penicillin-streptomycin were supplied from Gibco (Grand Island, NY, USA). Fetal bovine serum (FBS), RIPA buffer, protease inhibitors and Coomassie Plus™ Protein Assay Reagent were obtained from Thermo Scientific Company (Waltham, MA, USA). Guava Cell Nexin Reagent was purchased from Guava Technologies (Darmstadt, Germany). Nitrocellulose membrane and ECL reagent were supplied from GE Healthcare (Little Chalfont, UK). Gelatin, propidium iodide (PI) and 3-Methyladenine (3-MA) were obtained from Sigma (St. Louis, MO, USA). Antibodies specific to COX-2, and cyclin -D1 were purchased from Millipore (Darmstadt, Germany). Antibodies specific to β-actin, uPA, urokinase-type plasminogen activator receptor (uPAR), poly (ADP-ribose) polymerase (PARP), and p65 were purchased from Santa Cruz Biotechnology (Santa Cruz, CA, USA). Antibodies for the detection of ERK1/2, p38, JNK, c-Jun, and p65 were purchased from Cell Signaling Technology (Danvers, MA, USA). Matrigel was purchased from Becton Dickinson (Bedford, MA, USA).

### 4.2. Preparation of Proanthocyanidin-Rich Fraction from Red Rice Extract

Whole grains of red rice (Oryza sativa L.) collected from Doi Saket District (Chiang Mai, Thailand) were dehulled and polished in order to obtain the rice germ and bran using a rice de-husker and a rice milling machine (Kinetic (Hubei) Energy Equipment Engineering Co., Ltd., Wuhan, Hubei, China). A voucher specimen was certified by the herbarium at the Flora of Thailand, Faculty of Pharmacy, Chiang Mai University (voucher specimen no. 023148). 

Proanthocyanidin-rich fraction (PRFR) was prepared by following the previously reported protocol [20]. Briefly, 440 g red rice bran were soaked in 50% ethanol for 24 h. After that, the mixture was filtered to separate the ethanolic fractions. The ethanolic fractions were evaporated and partitioned with saturated butanol. The saturated butanol fractions were collected and evaporated to obtain the medium polar fractions. Next, the PRFR was prepared form the medium polar fractions by using Sephadex LH20 (GE Healthcare) chromatography (GE Healthcare Ltd., Little Chalfont, UK). The medium polar fractions (3.5 g) were dissolved in methanol and loaded onto a Sephadex LH-20 column. The fractions were sequentially eluted with solutions of 70% methanol, 30% methanol, and 70% acetone, respectively. Total contents of proanthocyanidins in each fraction were determined by vanillin assay. The fractions containing high concentrations of proanthocyanidins were pooled together and freeze-dried in order to obtain PRFR powder. The total amount of proanthocyanidins in the PRFR was 177.22 ± 16.66 mg catechin/g extract.

### 4.3. Cell Cultures

A549 lung adenocarcinoma cells were supplied by ATCC. The cells were cultured in DMEM supplemented with 100 U/mL penicillin, and 100 μg/mL streptomycin plus 10% FBS. The cultures were maintained in a humidified incubator with an atmosphere comprised of 95% air and 5% CO_2_ at 37 °C. For the PRFR treatment, PRFR was dissolved in DMSO and diluted with culture medium, for which the final concentration of DMSO was less than 0.1% (*v*/*v*).

### 4.4. Cell Viability Assay

The cell viability assay of PRFR against A549 lung adenocarcinoma cells was evaluated using trypan blue staining. Briefly, 2 × 10^4^ cells/well were seeded in a 24-well plate and incubated at 37 °C, 5% CO_2_ for 24 h in DMEM containing 10% FBS. After that, the cells were treated with or without various concentrations (0–200 μg/mL) of PRFR in DMEM containing 10% FBS for 24 h. At the end of the treatment, the percent of cell viability was also determined from counts of the cells suspended in the medium and counts of those cells removed from the plates by trypsinization. Equal parts of 0.4% trypan blue dye were added to the cell suspension in order to obtain a 1 to 2 ratio. The cell viability in each well was determined using Trypan blue dye and the values were compared with the controls.

### 4.5. Cell Cycle Arrest Assay

A549 cells were incubated with or without various concentrations of the proanthocyanidin-rich fraction (0–50 µg/mL) for 24 h. Then, the cell suspension was prepared on ice and stained with propidium iodide (PI) for 30 min in the dark. Cells were washed with cold PBS and resuspended in 500 μL. For cell cycle analysis, 1 × 10^4^ events were recorded and then analyzed with the BD FACScan^TM^ flow cytometer (BD Biosciences, San Jose, CA, USA).

### 4.6. Apoptosis Assay

A549 cells were incubated with or without various concentrations of PRFR (0–50 µg/mL) for 24 h. The cell suspension was then prepared and stained with annexin V and 7-amino actinomycin D (Guava Cell Nexin Reagent; Guava Technologies) for 20 min according to the Guava Nexin Assay protocol. Apoptosis was determined on a Guava PCA Instrument using Guava® Viacount^TM^ Software (Merck Ltd., Darmstadt, Germany).

### 4.7. Extraction of Nuclear and Whole-Cell Lysate

Whole-cell extraction was done to determine the expression levels of the invasive, apoptotic and survival proteins in the A549 cells. The cells were pretreated with various concentrations of PRFR for 4 h and treated with 25 ng/mL of TNF-α for 24 in DMEM medium to determine the levels of uPA, uPAR, COX-2, Survivin, cFLIPs, cyclin D, LC3B, caspase-8, and PARP-1 proteins. The levels of the MAPKs and Akt pathway proteins were determined from the cells treated with PRFR. After that, the cells were treated with TNF-α (25 ng/mL) for 15 min. The treated cells were then extracted using a RIPA lysis buffer containing protease inhibitors (1 mM PMSF, 10 μg/mL leupeptin, 10 μg/mL aprotinin) for 20 min on ice. The insoluble matter was removed by centrifugation at 12,000 rpm for 15 min at 4 °C, and the supernatant fraction (whole cell lysate) was collected and protein concentration was determined using Bradford protein assay. 

For the preparation of the nuclear extract fractions, after the A549 cells were treated with PRFR (0–15 μg/mL), TNF-α (25 ng/mL) was added to the cells and they were incubated for 1 h at 37 °C. The treated cells were then collected and the cell pellets were suspended with 50 μL of lysis buffer (10 mM HEPES, pH 7.9, 10 mM KC1, mM EDTA, 0.1 mM EGTA, 1 mM dithiothreitol, 0.5 mM phenylmethylsulfonyl -fluoride, 0.1 μg/mL leupeptin, 1 μg/mL aprotinin). The cells were allowed to swell on ice for 20 min, after which, 15 μL of 10% of Nonidet P-40 was added. The tubes were agitated on a vortex and centrifuged at 12,000 rpm for 5 min. The supernatant was collected and was representative of the cytoplasm extract. The nuclear pellets were suspended in ice-cold nuclear extraction buffer (20 mM HEPES, pH 7.9, 0.4 M NaCl, 1 mM EDTA, 1 mM EGTA, 1 mM dithiothreitol, 1 mM phenylmethylsulfonylfluoride, 2.0 μg/mL leupeptin, 2.0 μg/mL aprotinin) with an intermittent vortex for 30 min. The nuclear extract was centrifuged at 12,000 rpm for 10 min, and the supernatant was collected and used to determine the resulting yield of nuclear proteins.

### 4.8. Western Blotting Analysis 

The whole cell lysate or nuclear extractions were subjected to 10–12% SDS-PAGE. The proteins were transferred onto nitrocellulose membranes. The membranes were blocked with 5% non-fat dried milk protein in 0.5% TBS-tween. Thereafter, the membranes were further incubated overnight with the desired primary antibody at 4 °C followed by incubation with horseradish peroxidase conjugated secondary antibody. Bound antibodies were detected using the chemiluminescent detection system and then exposed to the X-ray film (GE Healthcare Ltd., Little Chalfont, U.K.). Equal values of protein loading were confirmed as each membrane was stripped and re-probed with an anti-β-actin antibody.

### 4.9. Monodansylcadaverine Staining 

The treated A549 cells were stained with 0.05 mM Monodansylcadaverin (MDC) in PBS for 30 min at 37 °C. The cells were washed three times with PBS to remove excess MDC. The visualization step employed a Carl Zeiss Microscopy GmbH (Carl Zeiss AG, Jena, Germany) with an excitation wavelength of 460–500 nm and an emission wavelength of 512–542 nm.

### 4.10. Statistical Analysis

All data are presented as mean ± standard deviation (S.D.) values. Statistical analysis was analyzed with Prism version 6.0 software GmbH (GraphPad Software, Inc. , San Diego, CA, USA) using one-way ANOVA with Dunnett’s test. Statistical significance was determined at * *p* < 0.05, ** *p* < 0.01, *** *p* < 0.001, or **** *p* < 0.0001.

## 5. Conclusions

PRFR was determined that could enhance TNF-α-induced A549 cell death by inducing autophagy and inhibiting cell invasion. PRFR suppressed TNF-α-induced the expression of survival, proliferation and invasive proteins. This was, at least in part, due to the reduced values of the MAPKs, Akt, NF-κB and AP-1 signaling pathways. Therefore, these findings provide important new evidence that can assist researchers to gain a better understanding of the anti-cancer activity of PRFR, which can facilitate further investigations into its potential for use in anti-cancer therapy.

## References

[1] Bray F., Ferlay J., Soerjomataram I., Siegel R.L., Torre L.A., Jemal A. (2018). Global cancer statistics 2018: GLOBOCAN estimates of incidence and mortality worldwide for 36 cancers in 185 countries. CA A Cancer J. Clin..

[2] Farbicka P., Nowicki A. (2013). Palliative care in patients with lung cancer. Contemp. Oncol. (Pozn).

[3] Pope C.A., Burnett R.T., Thun M.J., Calle E.E., Krewski D., Ito K., Thurston G.D. (2002). Lung cancer, cardiopulmonary mortality, and long-term exposure to fine particulate air pollution. JAMA.

[4] Beelen R., Hoek G., van den Brandt P.A., Goldbohm R.A., Fischer P., Schouten L.J., Armstrong B., Brunekreef B. (2008). Long-Term Exposure to Traffic-Related Air Pollution and Lung Cancer Risk. Epidemiology.

[5] Kim H.-B., Shim J.-Y., Park B., Lee Y.-J. (2018). Long-Term Exposure to Air Pollutants and Cancer Mortality: A Meta-Analysis of Cohort Studies. Int. J. Environ. Res. Public Health.

[6] Araújo A. (2016). Inflammation and Lung Cancer Oxidative Stress, ROS, and DNA Damage. React. Oxyg. Spec. Biol. Hum. Health.

[7] Multhoff G., Molls M., Radons J. (2012). Chronic inflammation in cancer development. Front. Immunol..

[8] Shang G.S., Liu L., Qin Y.W. (2017). IL-6 and TNF-alpha promote metastasis of lung cancer by inducing epithelial-mesenchymal transition. Oncol. Lett..

[9] Perez-Gracia J.L., Prior C., Guillén-Grima F., Segura V., Gonzalez A., Panizo A., Melero I., Grande-Pulido E., Gurpide A., Gil-Bazo I. (2009). Identification of TNF-α and MMP-9 as potential baseline predictive serum markers of sunitinib activity in patients with renal cell carcinoma using a human cytokine array. Br. J. Cancer.

[10] Devin A., Lin Y., Liu Z.G. (2003). The role of the death-domain kinase RIP in tumour-necrosis-factor-induced activation of mitogen-activated protein kinases. EMBO Rep..

[11] Sethi G., Sung B., Aggarwal B.B. (2008). TNF: A master switch for inflammation to cancer. Front. Biosci..

[12] Wang X., Lin Y. (2008). Tumor necrosis factor and cancer, buddies or foes?. Acta Pharm. Sin..

[13] Guicciardi M.E., Gores G.J. (2009). Life and death by death receptors. FASEB J..

[14] Van Herreweghe F., Festjens N., Declercq W., Vandenabeele P. (2010). Tumor necrosis factor-mediated cell death: To break or to burst, that’s the question. Cell. Mol. Life Sci..

[15] Harris J. (2011). Autophagy and cytokines. Cytokine.

[16] Safa A.R. (2013). Roles of c-FLIP in Apoptosis, Necroptosis, and Autophagy. J. Carcinog. Mutagen..

[17] Sahni S., Merlot A.M., Krishan S., Jansson P.J., Richardson D.R. (2014). Gene of the month: BECN1. J. Clin. Pathol..

[18] Gabetta B., Fuzzati N., Griffini A., Lolla E., Pace R., Ruffilli T., Peterlongo F. (2000). Characterization of proanthocyanidins from grape seeds. Fitoterapia.

[19] Limtrakul P., Yodkeeree S., Pitchakarn P., Punfa W. (2016). Anti-inflammatory effects of proanthocyanidin-rich red rice extract via suppression of MAPK, AP-1 and NF-κB pathways in Raw 264.7 macrophages. Nutr. Res. Pract..

[20] Pintha K., Yodkeeree S., Limtrakul P. (2015). Proanthocyanidin in red rice inhibits MDA-MB-231 breast cancer cell invasion via the expression control of invasive proteins. Biol. Pharm. Bull..

[21] Upanan S., Yodkeeree S., Thippraphan P., Punfa W., Wongpoomchai R., Limtrakul Dejkriengkraikul P. (2019). The Proanthocyanidin-Rich Fraction Obtained from Red Rice Germ and Bran Extract Induces HepG2 Hepatocellular Carcinoma Cell Apoptosis. Molecules.

[22] Tanida I., Ueno T., Kominami E. (2008). LC3 and Autophagy. Autophagosome and Phagosome.

[23] Koukourakis M.I., Kalamida D., Giatromanolaki A., Zois C.E., Sivridis E., Pouliliou S., Mitrakas A., Gatter K.C., Harris A.L. (2015). Autophagosome Proteins LC3A, LC3B and LC3C Have Distinct Subcellular Distribution Kinetics and Expression in Cancer Cell Lines. PLoS ONE.

[24] Gu L., Kelm M.A., Hammerstone J.F., Beecher G., Holden J., Haytowitz D., Gebhardt S., Prior R.L. (2004). Concentrations of proanthocyanidins in common foods and estimations of normal consumption. J. Nutr..

[25] Levine B., Yuan J. (2005). Autophagy in cell death: An innocent convict?. J. Clin. Investig..

[26] Gozuacik D., Kimchi A. (2004). Autophagy as a cell death and tumor suppressor mechanism. Oncogene.

[27] Sivaprasad U., Basu A. (2008). Inhibition of ERK attenuates autophagy and potentiates tumour necrosis factor-α-induced cell death in MCF-7 cells. J. Cell. Mol. Med..

[28] FBauvy D.-M.M.M.J. (2006). NF-kappaB activation represses tumor necrosis factor-alpha-induced autophagy. J. Biol. Chem..

[29] Zhu J., Cai Y., Xu K., Ren X., Sun J., Lu S., Chen J., Xu P. (2018). Beclin1 overexpression suppresses tumor cell proliferation and survival via an autophagy-dependent pathway in human synovial sarcoma cells. Oncol. Rep..

[30] Radeff-Huang J., Seasholtz T.M., Chang J.W., Smith J.M., Walsh C.T., Brown J.H. (2007). Tumor necrosis factor-α-stimulated cell proliferation is mediated through sphingosine kinase-dependent Akt activation and cyclin D expression. J. Biol. Chem..

[31] Gupta S.C., Kim J.H., Prasad S., Aggarwal B.B. (2010). Regulation of survival, proliferation, invasion, angiogenesis, and metastasis of tumor cells through modulation of inflammatory pathways by nutraceuticals. Cancer Metastasis Rev..

[32] Liu B., Qu L., Yan S. (2015). Cyclooxygenase-2 promotes tumor growth and suppresses tumor immunity. Cancer Cell Int..

[33] Pang L.Y., Hurst E.A., Argyle D.J. (2016). Cyclooxygenase-2: A role in cancer stem cell survival and repopulation of cancer cells during therapy. Stem Cells Int..

[34] Qiao Y., He H., Jonsson P., Sinha I., Zhao C., Dahlman-Wright K. (2016). AP-1 is a key regulator of proinflammatory cytokine TNFα-mediated triple-negative breast cancer progression. J. Biol. Chem..

[35] Balkwill F. (2006). TNF-α in promotion and progression of cancer. Cancer Metastasis Rev..

[36] Djavaheri-Mergny M., Amelotti M., Mathieu J., Besançon F., Bauvy C., Codogno P. (2007). Regulation of autophagy by NF-kappaB transcription factor and reactives oxygen species. Autophagy.

[37] Djavaheri-Mergny M., Amelotti M., Mathieu J., Besançon F., Bauvy C., Souquère S., Pierron G., Codogno P. (2006). NF-κB activation represses tumor necrosis factor-α-induced autophagy. J. Biol. Chem..

[38] Jang J.-Y., Lee J.-K., Jeon Y.-K., Kim C.-W. (2013). Exosome derived from epigallocatechin gallate treated breast cancer cells suppresses tumor growth by inhibiting tumor-associated macrophage infiltration and M2 polarization. BMC Cancer.

[39] Pratheeshkumar P., Sreekala C., Zhang Z., Budhraja A., Ding S., Son Y.-O., Wang X., Hitron A., Hyun-Jung K., Wang L. (2012). Cancer prevention with promising natural products: Mechanisms of action and molecular targets. Anti-Cancer Agents Med. Chem. (Former. Curr. Med. Chem. Anti-Cancer Agents).

[40] Noguchi M., Hirata N., Suizu F. (2014). The links between AKT and two intracellular proteolytic cascades: Ubiquitination and autophagy. Biochim. Biophys. Acta (Bba) Rev. Cancer.

